# Real-World Safety and Circuit Outcomes of Protocolized Divided-Dose Enoxaparin During Continuous Renal Replacement Therapy Without Routine Anti-Xa Monitoring: A Single-Center Competing-Risk Cohort Study

**DOI:** 10.3390/jcm15145345

**Published:** 2026-07-08

**Authors:** Hasan Burak Toprak, Gürhan Taşkın, Muhammet Alperen Bayrak, Mahir Sallan, Oya Kocakanat, Mete Erdemir, Levent Yamanel

**Affiliations:** Department of Intensive Care, Gülhane Training and Research Hospital, Ankara 06010, Türkiye

**Keywords:** continuous renal replacement therapy, CRRT, enoxaparin, low-molecular-weight heparin, circuit clotting, regional citrate anticoagulation, competing risk, bleeding, intensive care

## Abstract

**Background/Objectives:** Regional citrate anticoagulation (RCA) is guideline-preferred for continuous renal replacement therapy (CRRT), yet implementation requires expertise, calcium protocols, and reliable monitoring. Evidence for standardized low-molecular-weight heparin strategies without routine anti-Xa monitoring remains limited. **Methods**: We retrospectively analyzed adult ICU patients receiving protocolized divided-dose enoxaparin during CRRT from January 2020 to December 2024. Enoxaparin 1.5 mg/kg/24 h was divided into six equal prefilter doses every 4 h. The primary outcome was circuit clotting; death and hemodynamic instability were treated as competing termination events. Safety endpoints were ISTH major bleeding, clinically relevant non-major bleeding (CRNMB), minor bleeding, thrombosis, and suspected heparin-induced thrombocytopenia (HIT). **Results**: The cohort included 200 patients and 223 CRRT runs, contributing 8829.8 CRRT-hours. Median run duration was 35.0 h (IQR, 27.2–52.1). Circuit clotting occurred in 31 runs (13.9%; 95% CI, 9.6–19.1), equivalent to 0.35 events per 100 CRRT-hours. Kaplan–Meier clotting-free survival at 48 h was 89.8% (95% CI, 84.3–95.2), and the competing-risk cumulative incidence of clotting was 9.0%. Any classified bleeding occurred in 10 runs (4.5%; 95% CI, 2.2–8.1), including one ISTH major bleeding event and one CRNMB event. No thrombotic or HIT events were identified. In a prespecified four-variable Cox model with patient-level cluster-robust standard errors, no predictor was significantly associated with clotting. **Conclusions**: In a citrate-unavailable or citrate-not-routinely-implemented ICU setting, this standardized divided-dose enoxaparin protocol showed low observed major/clinically relevant bleeding rates and acceptable clotting-free circuit performance. Prospective comparative evaluation is warranted.

## 1. Introduction

Continuous renal replacement therapy (CRRT) is a core organ-support modality for critically ill patients with acute kidney injury, severe fluid overload, and hemodynamic instability. Because extracorporeal blood exposure promotes activation of coagulation pathways, anticoagulation is often required to prevent premature filter loss and treatment interruption. Current kidney-support guidance emphasizes that the choice of anticoagulation should balance circuit patency with bleeding risk, patient-level contraindications, and local expertise [1].

Regional citrate anticoagulation (RCA) has become the best-supported strategy in many CRRT populations. Randomized data and meta-analyses suggest that citrate can prolong filter life and reduce bleeding compared with systemic heparin-based approaches, although effects on patient-centered outcomes are less consistent [2,3,4,5]. These advantages explain why citrate is frequently considered the preferred CRRT anticoagulant when it can be delivered safely and reliably.

However, citrate is not simply a pharmacologic substitution for heparin. It is a protocol-intensive ICU process requiring calcium-free or citrate-compatible replacement fluids, calcium infusion algorithms, frequent electrolyte and acid–base monitoring, recognition of citrate accumulation, and a trained multidisciplinary team. Reviews and practice surveys continue to show substantial heterogeneity in CRRT anticoagulation, reflecting differences in resources, experience, and local implementation [6,7,8,9]. Therefore, real-world ICUs without routine citrate implementation still require reproducible alternative anticoagulation strategies.

Low-molecular-weight heparins (LMWHs), including enoxaparin, are attractive in some settings because they have predictable pharmacokinetics in non-CRRT contexts and can be operationalized with simple dosing schedules. Nevertheless, evidence during CRRT is sparse and heterogeneous. Prior reports include small randomized comparisons, audits, dalteparin- or enoxaparin-based protocols, and more recent RCA-versus-LMWH cohorts in specialized populations such as hyperlactatemia and liver failure [10,11,12,13,14,15,16,17]. These studies do not fully resolve whether a standardized divided-dose enoxaparin protocol can be used safely in routine ICU CRRT practice without routine anti-Xa monitoring.

Beyond anticoagulant choice, circuit life is influenced by catheter site and function, blood flow, filtration fraction, patient inflammation, thrombocytopenia, shock, hemoconcentration, and technical interruptions. Recent systematic and prediction-model literature emphasizes that clotting is a multifactorial outcome rather than a direct drug-effect endpoint alone [18,19,20]. Consequently, a methodologically defensible CRRT anticoagulation cohort should distinguish patient-level and run-level denominators, account for repeated runs, and use time-to-event and competing-risk methods.

The present study evaluates a long-running institutional protocol that administered enoxaparin into the prefilter limb of the CRRT circuit at a total daily dose of 1.5 mg/kg/24 h divided into six equal prefilter doses every 4 h. To our knowledge, within the limited LMWH-focused CRRT literature, this is one of the larger run-level cohorts evaluating a standardized divided-dose enoxaparin protocol during CRRT without routine anti-Xa monitoring. We aimed to describe real-world circuit and safety outcomes, with conservative interpretation, in a citrate-unavailable or citrate-not-routinely-implemented ICU setting. We specifically avoided claims of superiority or equivalence to citrate and focused instead on reproducibility, bleeding safety, clotting-free circuit performance, and competing-risk circuit analysis.

## 2. Materials and Methods

### 2.1. Study Design and Setting

This was a single-center retrospective cohort study of adult ICU patients receiving CRRT with protocolized divided-dose enoxaparin anticoagulation between January 2020 and December 2024. The study was designed as a real-world protocol evaluation rather than as a comparative effectiveness study.

Ethical approval was obtained from the University of Health Sciences, Gülhane Scientific Research Ethics Committee (decision no. 2026-193; meeting no. 2026/05; 12 May 2026) and the requirement for individual informed consent was waived for this retrospective analysis of de-identified routine clinical data.

### 2.2. Study Population and Analytic Unit

Eligible observations were adult ICU CRRT runs anticoagulated according to the institutional enoxaparin protocol. The primary analytic unit was the CRRT run because circuit clotting, circuit termination, and filter survival are run-level outcomes. Patient-level characteristics were summarized once per unique patient. Repeated CRRT runs within the same patient were retained in the primary run-level analysis, and repeated-run dependence was addressed by patient-level cluster-robust standard errors and first-run-only sensitivity analysis.

**Figure 1:**
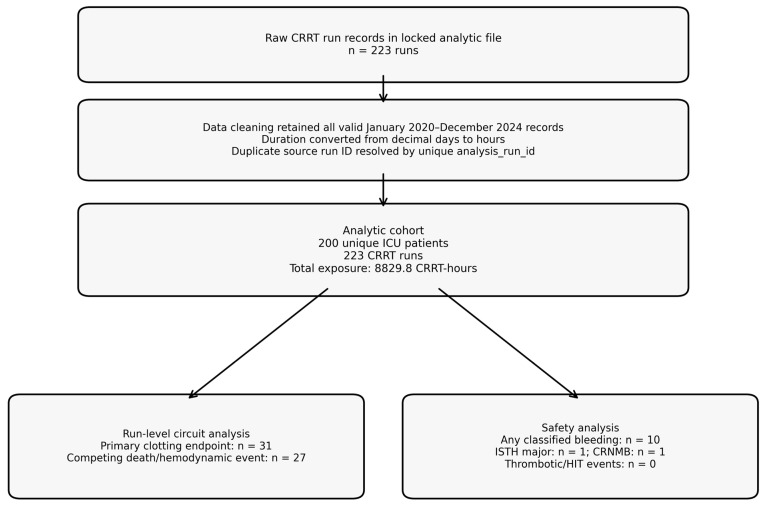
Study flowchart and analytic denominator. The figure summarizes the cleaned analytic cohort, patient-level and run-level denominators, and the separation of circuit and safety analyses.

The cohort flow is shown in Figure 1. The final cleaned dataset included 200 unique patients and 223 CRRT runs. The original run identifier was preserved for traceability; a unique analysis run identifier was assigned to ensure unambiguous run-level analysis.

### 2.3. Institutional Enoxaparin Protocol

The institutional protocol administered enoxaparin into the prefilter limb of the CRRT circuit. The total daily dose was 1.5 mg/kg/24 h, divided into six equal doses administered every 4 h. The strategy was intended to provide reproducible circuit anticoagulation using an LMWH-based schedule in a setting where citrate was not routinely implemented. Routine systemic anti-Xa monitoring was not part of the protocol. The protocol was designed for operational reproducibility in an ICU where regional citrate anticoagulation was not routinely implemented and rapid-turnaround anti-Xa assays were not routinely available. Although routine anti-Xa monitoring was not performed, patients underwent structured bedside safety surveillance as part of standard ICU care, including daily clinical assessment for overt bleeding, serial hemoglobin and platelet-count monitoring, review of transfusion requirements, and assessment of invasive lines, drains, gastrointestinal output, and other potential bleeding sites. Enoxaparin was withheld when clinically relevant bleeding was suspected or documented and was reassessed before re-initiation. No concomitant unfractionated heparin or citrate anticoagulation was recorded in the analytic runs.

The protocol section is intentionally detailed because interpretation of LMWH-based CRRT studies depends heavily on dose, administration site, monitoring approach, and termination rules. The present analysis should therefore be read as an evaluation of a specific standardized strategy rather than as a general endorsement of unmonitored LMWH use in all CRRT patients.

### 2.4. CRRT Procedure, Run Duration, and Termination Taxonomy

All analyzed runs used continuous venovenous hemodialysis (CVVHD). CRRT was delivered with Fresenius multiFiltrate PRO and multiFiltrate devices (Fresenius Medical Care, Bad Homburg, Germany). All circuits used a standard high-flux polysulfone hemofilter (AV600S) with a 12-Fr double-lumen venous catheter. Blood flow was not assigned by a fixed anticoagulation-specific algorithm but was individualized according to vascular-access performance, achievable circuit pressures, catheter function, and hemodynamic tolerance; lower rates were used when catheter dysfunction, excessive negative access pressures, or hemodynamic instability limited higher flows. Run duration was measured from CRRT initiation to circuit termination. During data cleaning, the source duration variable was found to be stored as decimal days despite an hour-like label; therefore, cleaned duration was calculated by multiplying the raw value by 24 and cross-checking against available start and end timestamps.

Circuit termination was classified as clotting, death, hemodynamic instability, elective termination, or technical termination. The primary circuit event was clotting, defined by normalized circuit end-reason equal to clotting. Death and hemodynamic instability were prespecified as competing events because they preclude subsequent observation of clotting in the same run. Elective termination denoted a planned, protocol-driven discontinuation not caused by clotting, death, hemodynamic instability, or technical failure, encompassing scheduled filter replacement at the protocol-defined 72 h limit, discontinuation after achievement of the prescribed treatment target, and cessation of CRRT following clinical or renal improvement; these subreasons were not consistently coded as separate structured variables and could not be quantified individually. Elective and technical terminations were treated as censoring events in time-to-event analyses.

### 2.5. Bleeding, Thrombosis, and HIT Adjudication

Bleeding was adjudicated as ISTH major bleeding, ISTH clinically relevant non-major bleeding (CRNMB), or minor bleeding. ISTH major bleeding and CRNMB definitions were used to improve comparability with anticoagulation literature [21,22]. A hemoglobin decline or red-cell transfusion note without clinically evident bleeding was not counted as a primary bleeding endpoint; such observations were retained as unadjudicated safety notes.

Thrombotic events and suspected heparin-induced thrombocytopenia (HIT) were evaluated from the available thrombotic-event and clinical documentation fields. Given the low event count, bleeding and thrombotic safety outcomes were summarized using event counts, exact 95% confidence intervals, and event-rate estimates per 100 CRRT-hours. Bleeding regression was not fitted because the number of classified bleeding events was too small for stable multivariable modeling.

### 2.6. Statistical Analysis

Continuous variables are summarized as median and interquartile range (IQR), and categorical variables as counts and percentages. Exact binomial 95% confidence intervals were calculated for key proportions. Event rates per 100 CRRT-hours were estimated using total observed CRRT exposure as the denominator. Kaplan–Meier methods estimated clotting-free circuit survival with non-clotting terminations censored. Missing data were not imputed, and all available observations were used for each variable; the variables used in the time-to-event and regression analyses were complete, and no imputation or missing-data sensitivity analysis was performed for the descriptive baseline characteristics.

Competing-risk analysis estimated the cumulative incidence function (CIF) for circuit clotting while treating death or hemodynamic instability as competing events. A Fine–Gray subdistribution model was used as a competing-risk approximation for predictor analysis [23]. Because there were 31 primary clotting events, multivariable modeling was restricted to four clinically prespecified binary predictors: femoral catheter, platelet count <100 × 10^3^/µL, septic shock, and therapeutic anticoagulation indication. The main cause-specific Cox model used patient-level cluster-robust sandwich standard errors to address repeated runs within the same patient. Sensitivity analyses used the original clotting flag, first CRRT run per patient, and exclusion of technical terminations.

## 3. Results

### 3.1. Patient-Level Baseline Characteristics

The final cohort included 200 unique adult ICU patients. Baseline patient characteristics are presented in Table 1. The cohort was elderly and severely ill, with median age 72.5 years, median APACHE II score 27, and median SOFA score 7. Sepsis was present in 163/200 patients (81.5%), septic shock in 114/200 (57.0%), and vasopressor use in 124/200 (62.0%). Mechanical ventilation was recorded in 120/200 patients (60.0%). Baseline chronic kidney disease was common, affecting 97/200 patients (48.5%). Cirrhosis, with or without other chronic liver disease, was recorded in 10/200 patients (5.0%). These features indicate that the protocol was applied in a high-acuity ICU population rather than a low-risk dialysis cohort.

### 3.2. CRRT Run Characteristics

Run-level CRRT characteristics are summarized in Table 2. The analysis included 223 CRRT runs with a total observed exposure of 8829.8 CRRT-hours. All runs used CVVHD. Median run duration was 35.0 h (IQR, 27.2–52.1), median blood flow was 140 mL/min, and the median prescribed effluent dose was 30 mL/kg/h. Internal jugular venous catheters were used in 122/223 runs (54.7%), femoral venous catheters in 96/223 (43.0%), and subclavian venous catheters in 5/223 (2.2%). No run had recorded concomitant unfractionated heparin or citrate anticoagulation.

Circuit termination was elective in 160/223 runs (71.7%), clotting-related in 31/223 (13.9%), death-related in 17/223 (7.6%), hemodynamic-instability-related in 10/223 (4.5%), and technical in 5/223 (2.2%). Therefore, death or hemodynamic instability accounted for 27 competing terminations in the competing-risk framework. The relatively short median run duration should not be interpreted as early circuit failure: elective terminations had a median duration of 36.4 h (IQR, 27.9–53.8), whereas runs ending in death or hemodynamic instability were necessarily shorter (median 18.0 h and 23.5 h, respectively), and runs terminating due to clotting ran longer (median 50.0 h).

### 3.3. Circuit and Safety Outcomes

Primary circuit and safety outcomes are shown in Table 3. Using the cleaned end-reason definition, circuit clotting occurred in 31/223 runs (13.9%; exact 95% CI, 9.6–19.1), corresponding to 0.35 clotting events per 100 CRRT-hours (95% CI, 0.24–0.50). The original clotting flag identified 30 events, reflecting one discordant row in which the circuit end reason was clotting but the original event flag was not set. This difference was handled by using the cleaned end-reason endpoint as the primary definition and the original event flag as a sensitivity analysis.

Any classified bleeding occurred in 10/223 runs (4.5%; exact 95% CI, 2.2–8.1), with one ISTH major bleeding event, one CRNMB event, and eight minor bleeding events. Three additional observations had hemoglobin-drop or transfusion notes without a clinically adjudicated bleeding flag and were not counted as primary bleeding events. No thrombotic or HIT events were identified. The safety endpoint distribution is summarized graphically in the safety endpoint figure.

### 3.4. Time-to-Event and Competing-Risk Circuit Outcomes

**Figure 2:**
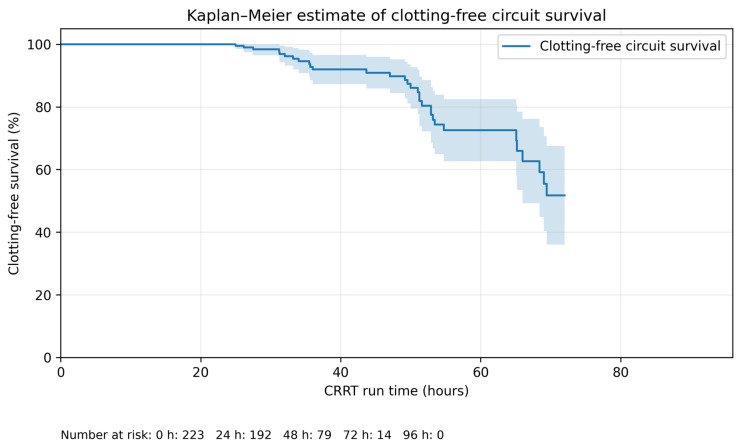
Kaplan–Meier clotting-free circuit survival. Non-clotting terminations were censored, so the curve estimates time to circuit clotting rather than uninterrupted CRRT continuation for any reason.

**Figure 3:**
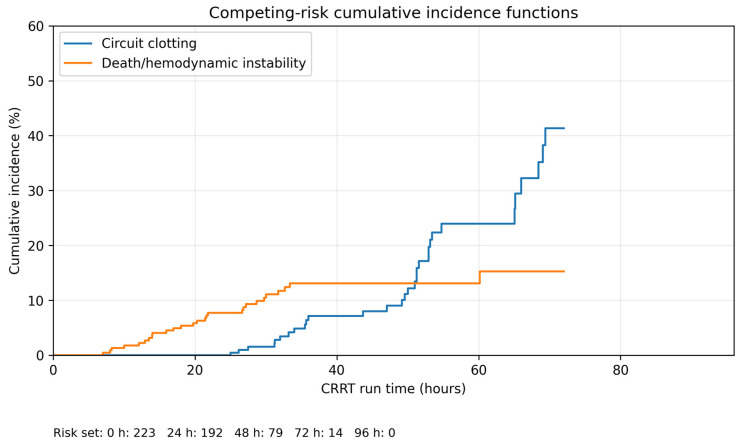
Competing-risk cumulative incidence curves. Circuit clotting is shown as the event of interest; death or hemodynamic instability is shown as the competing event. Elective and technical terminations were censored. Abbreviations: CRRT, continuous renal replacement therapy; CIF, cumulative incidence function.

**Figure 4:**
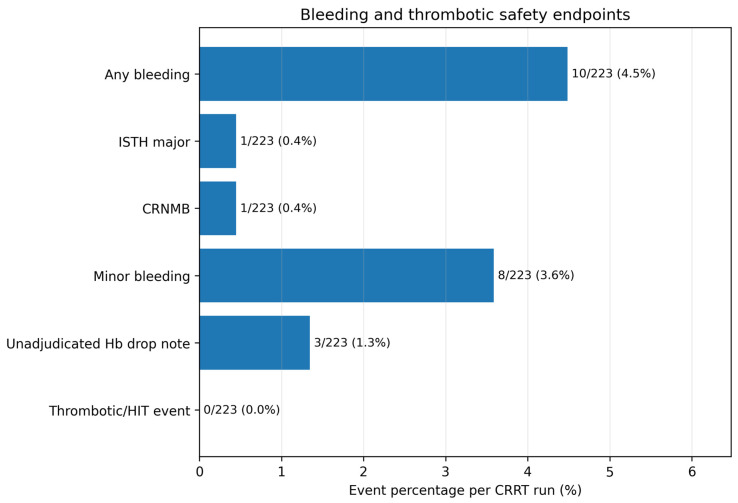
Bleeding and thrombotic safety endpoint summary. Bars show event percentages using 223 CRRT runs as the denominator. Hemoglobin-drop notes without clinically evident bleeding were retained as unadjudicated notes and were not counted as primary bleeding events. Abbreviations: CRNMB, clinically relevant non-major bleeding; HIT, heparin-induced thrombocytopenia; ISTH, International Society on Thrombosis and Haemostasis; CRRT, continuous renal replacement therapy.

Kaplan–Meier and competing-risk landmarks are presented in Table 4. Kaplan–Meier clotting-free survival was 100.0% at 24 h, 89.8% (95% CI, 84.3–95.2) at 48 h, and 51.8% (95% CI, 36.0–67.6) at 72 h. The corresponding CIF for clotting was 9.0% at 48 h and 41.3% at 72 h when death or hemodynamic instability were treated as competing events. Figure 2 displays the Kaplan–Meier curve, while Figure 3 shows the competing cumulative incidence functions. The safety endpoint distribution is shown in Figure 4.

The difference between crude observed 48 h filter continuation and Kaplan–Meier clotting-free survival is important. Only 79/223 runs were still at risk at 48 h, but many non-clotting terminations reflected elective discontinuation, death, hemodynamic instability, or technical discontinuation rather than circuit clotting. Time-to-event methods therefore better isolate clotting as a circuit endpoint than crude uninterrupted treatment duration alone. Estimates at the 72 h landmark are based on only 14 runs still at risk and are therefore imprecise; they are reported for completeness and should be interpreted with caution.

### 3.5. Prespecified Cox and Competing-Risk Models

Model results are shown in Table 5. The prespecified four-variable cause-specific Cox model used patient-level cluster-robust standard errors and included 31 clotting events across 223 runs and 200 patient clusters. No predictor reached conventional statistical significance. Femoral catheter use had a hazard ratio (HR) of 1.510 (95% CI, 0.604–3.776; *p* = 0.378), platelet count <100 × 10^3^/µL had an HR of 1.180 (95% CI, 0.533–2.613; *p* = 0.683), septic shock had an HR of 0.835 (95% CI, 0.335–2.082; *p* = 0.698), and therapeutic anticoagulation indication had an HR of 1.655 (95% CI, 0.689–3.977; *p* = 0.260).

Fine–Gray subdistribution estimates were broadly consistent with the cause-specific model. Therapeutic anticoagulation indication showed the largest point estimate in the subdistribution model (sHR, 2.117; 95% CI, 0.866–5.173; *p* = 0.100), but this remained imprecise and hypothesis-generating. Given the event count, no expanded 8–9 variable model was fitted.

### 3.6. Sensitivity Analyses

Sensitivity analyses are summarized in Table 6. Results were stable when the original event_filter_clotting flag was used instead of the cleaned end-reason endpoint, with 30 clotting events and an event rate of 0.34 per 100 CRRT-hours. Restricting the dataset to the first CRRT run per patient yielded 25 events among 200 runs and a similar event rate of 0.32 per 100 CRRT-hours. Excluding technical terminations also had minimal impact, with 31 events among 218 runs and a 48 h clotting CIF of 9.1%. These analyses support the robustness of the primary conclusion to endpoint discordance, repeated runs, and short technical terminations.

## 4. Discussion

In this real-world ICU cohort, protocolized divided-dose prefilter enoxaparin during CRRT showed acceptable clotting-free circuit performance together with a low observed rate of major and clinically relevant non-major bleeding. The analysis used run-level denominators, patient-level cluster-robust model variance, and competing-risk methods to avoid overstating circuit performance.

The most important interpretive boundary is that this study does not compare enoxaparin with citrate, unfractionated heparin, or no anticoagulation. Therefore, the results should not be interpreted as showing equivalence, non-inferiority, or superiority to RCA. Rather, the study addresses a narrower practical question: what circuit and safety outcomes were observed after long-term real-world use of a standardized LMWH-based CRRT protocol in an ICU where citrate was not routinely implemented?

RCA remains the best-supported anticoagulation strategy in many CRRT populations. Observational and protocol-change studies have generally found improved circuit performance after citrate implementation compared with systemic heparin or non-citrate strategies, although protocol complexity and patient selection remain important [24,25,26,27]. The present findings therefore complement, rather than challenge, the citrate literature. They are most relevant to ICUs that cannot reliably implement citrate or require an interim standardized alternative.

Placed alongside the existing literature, our observed clotting burden (13.9% of runs; 0.35 events per 100 CRRT-hours; 89.8% Kaplan–Meier clotting-free survival at 48 h) appears broadly compatible with the heterogeneous results reported for non-citrate and LMWH-based CRRT anticoagulation. Randomized and meta-analytic data indicate that regional citrate generally prolongs filter life and reduces bleeding relative to systemic heparin [2,3,4,5], whereas the smaller LMWH/enoxaparin literature—including randomized, audit, and cohort reports—describes heterogeneous circuit performance and safety across differing populations and protocols [10,11,12,13,14,15,16,17]. Direct numeric comparison is limited by substantial differences in study populations, circuit-termination definitions, filter-change policies, CRRT prescriptions, and the statistical handling of non-clotting terminations; we therefore frame this comparison qualitatively rather than as a head-to-head benchmark.

Implementation complexity is a central reason why anticoagulation protocols vary across ICUs. Citrate requires correct dialysate/replacement-fluid selection, calcium replacement, metabolic monitoring, and staff familiarity. Reviews of citrate implementation and liver-failure data emphasize that patient selection and protocolized monitoring are essential, particularly when citrate metabolism may be impaired [28,29,30]. This background supports the clinical rationale for reporting non-citrate real-world protocols transparently, provided claims remain conservative.

Cost and resource considerations also matter. Comparative economic analyses and high-bleeding-risk cohorts show that anticoagulation strategy is determined not only by biological efficacy; it is also shaped by nursing workload, monitoring burden, availability of fluids, local procurement, and clinician confidence [31,32,33,34,35]. An enoxaparin protocol that can be reproduced with a fixed daily dose divided every 4 h may be operationally feasible in settings without citrate infrastructure, but feasibility does not eliminate the need for careful bleeding surveillance.

A divided-dose (every 4 h) prefilter schedule was used rather than a continuous LMWH infusion for reasons of institutional operational simplicity, as it is straightforward to prescribe and reproduce on a fixed weight-based basis and can be readily withheld for procedures or bleeding; a continuous infusion may, however, yield more stable anti-Xa concentrations, and this trade-off is acknowledged. Enoxaparin is predominantly renally cleared, and its elimination is reduced in severe renal impairment, raising the theoretical possibility of accumulation during prolonged CRRT. Although the observed number of major and clinically relevant bleeding events was low, this does not exclude subclinical drug accumulation or establish comparative safety; the risk could not be directly assessed or excluded, and the protocol instead relied on a fixed, non-escalating dose and structured clinical bleeding surveillance, which did not substitute for anti-Xa measurement. Unfractionated heparin is another widely available non-citrate option, with the practical advantages of a short half-life, easy titration, and reversibility with protamine, although it carries its own bleeding and heparin-induced thrombocytopenia considerations; our data do not compare these strategies, and the choice should be individualized to local resources and patient bleeding risk.

The observed clotting outcomes are clinically plausible in light of the multifactorial nature of CRRT circuit failure. Catheter performance, blood flow, treatment interruptions, filter characteristics, shock physiology, inflammation, and hemoconcentration may all contribute to filter loss. Accordingly, raw circuit run duration reflects these multifactorial influences and cannot be attributed to anticoagulant efficacy alone. The absence of a strong independent predictor in the four-variable model should not be interpreted as proof that these factors are unimportant. Rather, with only 31 clotting events, the study was underpowered for definitive predictor discovery. The model was deliberately restricted to avoid overfitting.

The safety signal is notable but should be interpreted cautiously. The low number of major/clinically relevant bleeding events is reassuring, especially because patients were elderly and severely ill, but retrospective safety adjudication can miss subtle or incompletely documented bleeding. The single ISTH major event also demonstrates that clinically important bleeding can occur under this protocol. The analysis appropriately avoided bleeding regression because 10 classified bleeding events, including only one ISTH major event and one CRNMB event, were insufficient for stable multivariable inference.

The competing-risk analysis adds methodological value. In ICU CRRT, circuit discontinuation due to death or hemodynamic instability is common and prevents later observation of clotting in that run. Treating these events as simple censoring can overstate or distort the probability of clotting. The 48 h CIF for clotting was 9.0%, while competing death/hemodynamic instability reached 13.1%, illustrating the need to separate filter failure from patient deterioration. This distinction is particularly important in high-mortality ICU cohorts and aligns with increasing interest in CRRT circuit outcomes beyond crude filter lifespan [36,37].

Because ICU mortality was high (78%), death or hemodynamic instability terminated a substantial proportion of circuits before a clotting event could be observed; these outcomes were treated as competing events in the competing-risk analysis and were censored in the Kaplan–Meier analysis. Longer-duration estimates therefore increasingly reflect a selected, survival-enriched subset of patients whose circuits remained under observation, and the findings may not generalize to lower-acuity cohorts. A therapeutic anticoagulation indication was present in 32% of patients; however, this variable reflected the clinical indication rather than a standardized concurrent systemic anticoagulant regimen, and no concomitant unfractionated heparin was recorded in the analyzed runs, so our data cannot determine how the prefilter enoxaparin protocol should be combined with additional systemic anticoagulation.

This study has several strengths. It evaluates a clearly specified institutional anticoagulation protocol, includes consecutive real-world CRRT runs within the prespecified January 2020–December 2024 study window, distinguishes patient-level and run-level denominators, uses structured bleeding definitions, handles repeated runs with patient-level cluster-robust standard errors, and applies competing-risk methodology. The sensitivity analyses showed that the main findings were not materially changed by the one-row clotting endpoint discordance, first-run-only restriction, or exclusion of technical terminations.

The limitations are also important. The design was retrospective and single-center. There was no concurrent citrate, unfractionated heparin, or no-anticoagulation control group. Routine anti-Xa levels were not available, preventing assessment of drug accumulation or dose–response. Residual confounding is unavoidable, and the event count limited predictor modeling. Documentation of minor bleeding and thrombotic events may be incomplete. Finally, because all runs used CVVHD and no citrate was recorded, the findings may not generalize to centers using different CRRT modalities, membranes, replacement-fluid strategies, or anticoagulation monitoring protocols. In addition, because enoxaparin is predominantly renally cleared and the cohort consisted of critically ill patients requiring CRRT, low-molecular-weight heparin accumulation cannot be excluded; in the absence of anti-Xa monitoring it could not be detected. Retrospective designs also tend to under-ascertain thrombotic events, so the absence of documented thrombotic or HIT events should be interpreted as an absence of recorded events rather than proof that no such risk exists.

## 5. Conclusions

In a real-world ICU cohort with 200 patients and 223 CRRT runs, protocolized divided-dose prefilter enoxaparin was associated with acceptable clotting-free circuit performance and low observed rates of major and clinically relevant non-major bleeding. The study supports the feasibility of a standardized LMWH-based CRRT anticoagulation strategy in settings where citrate is unavailable or not routinely implemented. Because the cohort lacked a comparator and routine anti-Xa monitoring, the findings should be considered hypothesis-generating and should prompt prospective, multicenter, comparator-controlled evaluation rather than definitive practice change.

## Figures and Tables

**Table 1:** Baseline patient characteristics. Continuous variables are reported as median (interquartile range), and categorical variables are reported as n/N (%). The denominator is unique patients.

Characteristic	Overall Cohort (N = 200)	Summary Type
Age, years	72.5 (61.8–81.0)	Median (IQR)
Male sex	116/200 (58.0%)	n/N (%)
Body mass index, kg/m^2^	24.8 (22.9–27.5)	Median (IQR)
APACHE II score at CRRT start	27 (22–33)	Median (IQR)
SOFA score at CRRT start	7 (5–9)	Median (IQR)
Sepsis (Sepsis-3)	163/200 (81.5%)	n/N (%)
Septic shock	114/200 (57.0%)	n/N (%)
Chronic kidney disease at baseline	97/200 (48.5%)	n/N (%)
Cirrhosis/chronic liver disease	10/200 (5.0%)	n/N (%)
Malignancy	52/200 (26.0%)	n/N (%)
Recent major surgery within 7 days	6/200 (3.0%)	n/N (%)
Active bleeding at CRRT start	8/200 (4.0%)	n/N (%)
Mechanical ventilation	120/200 (60.0%)	n/N (%)
Vasopressor use	124/200 (62.0%)	n/N (%)
Norepinephrine-equivalent dose, µg/kg/min	0.09 (0.00–0.40)	Median (IQR)
Lactate at CRRT start, mmol/L	2.0 (1.3–3.6)	Median (IQR)
Hemoglobin, g/dL	9.5 (8.4–10.9)	Median (IQR)
Platelet count, 10^3^/µL	178 (120–264)	Median (IQR)
INR	1.30 (1.20–1.60)	Median (IQR)
aPTT, seconds	33.2 (28.8–41.0)	Median (IQR)
Fibrinogen, mg/dL	385 (255–506)	Median (IQR)
Albumin, g/dL	2.5 (2.2–3.0)	Median (IQR)
Therapeutic anticoagulation indication	64/200 (32.0%)	n/N (%)
ICU mortality	156/200 (78.0%)	n/N (%)
28-day mortality	125/200 (62.5%)	n/N (%)

Note: APACHE, Acute Physiology and Chronic Health Evaluation; CKD, chronic kidney disease; CRRT, continuous renal replacement therapy; ICU, intensive care unit; IQR, interquartile range; SOFA, Sequential Organ Failure Assessment.

**Table 2:** CRRT run characteristics and circuit termination taxonomy. The table summarizes modality, duration, vascular access, prescription parameters, and end-reason categories used for censoring and competing-risk analysis.

Characteristic	Overall Runs (N = 223)	Summary Type
CRRT runs analyzed	223	Count
Run duration, hours	35.0 (27.2–52.1)	Median (IQR)
CVVHD modality	223/223 (100.0%)	n/N (%)
Hemofilter (membrane)	AV600S (high-flux polysulfone)	Descriptive
Blood flow, mL/min	140 (120–150)	Median (IQR)
Effluent dose prescribed, mL/kg/h	30 (30–35)	Median (IQR)
Femoral venous catheter	96/223 (43.0%)	n/N (%)
Internal jugular venous catheter	122/223 (54.7%)	n/N (%)
Subclavian venous catheter	5/223 (2.2%)	n/N (%)
Filter survived 24 h	192/223 (86.1%)	n/N (%)
Filter survived 48 h	79/223 (35.4%)	n/N (%)
Concomitant UFH	0/223 (0.0%)	n/N (%)
Citrate used	0/223 (0.0%)	n/N (%)
Circuit end reason: Elective	160/223 (71.7%)	n/N (%)
Circuit end reason: Clotting	31/223 (13.9%)	n/N (%)
Circuit end reason: Death	17/223 (7.6%)	n/N (%)
Circuit end reason: Hemodynamic Instability	10/223 (4.5%)	n/N (%)
Circuit end reason: Technical	5/223 (2.2%)	n/N (%)

Note: CVVHD, continuous venovenous hemodialysis; CRRT, continuous renal replacement therapy; IQR, interquartile range; UFH, unfractionated heparin.

**Table 3:** Primary circuit and safety outcomes. Percentages use 223 CRRT runs as the denominator. Event rates use 8829.8 total CRRT-hours as the exposure denominator.

Outcome	Events	Denominator	Estimate	Exact 95% CI	Rate per 100 CRRT-Hours (95% CI)
Total CRRT exposure, hours			8829.8		
Circuit clotting (primary endpoint; cleaned end-reason definition)	31	223	31/223 (13.9%)	9.6–19.1%	0.35 (0.24–0.50)
Circuit clotting (original event_filter_clotting flag)	30	223	30/223 (13.5%)	9.3–18.6%	0.34 (0.23–0.49)
Competing circuit termination: death or hemodynamic instability	27	223	27/223 (12.1%)	8.1–17.1%	0.31 (0.20–0.44)
Any adjudicated bleeding	10	223	10/223 (4.5%)	2.2–8.1%	0.11 (0.05–0.21)
ISTH major bleeding	1	223	1/223 (0.4%)	0.0–2.5%	0.01 (0.00–0.06)
ISTH clinically relevant non-major bleeding	1	223	1/223 (0.4%)	0.0–2.5%	0.01 (0.00–0.06)
Minor bleeding	8	223	8/223 (3.6%)	1.6–6.9%	0.09 (0.04–0.18)
Unadjudicated Hb-drop/transfusion note without bleeding flag	3	223	3/223 (1.3%)	0.3–3.9%	0.03 (0.01–0.10)
Thrombotic/HIT events	0	223	0/223 (0.0%)	0.0–1.6%	0.00 (0.00–0.04)

Note: CI, confidence interval; CRNMB, clinically relevant non-major bleeding; CRRT, continuous renal replacement therapy; HIT, heparin-induced thrombocytopenia; ISTH, International Society on Thrombosis and Haemostasis.

**Table 4:** Time-to-event and competing-risk landmarks for circuit clotting. Kaplan–Meier estimates censor non-clotting terminations, whereas CIF estimates treat death and hemodynamic instability as competing circuit-termination events.

Time Landmark	At Risk	KM Clotting-Free Survival (95% CI)	CIF Clotting	CIF Death/Hemodynamic	Event-Free Survival
24 h	192	100.0% (100.0–100.0)	0.0%	7.7%	92.3%
48 h	79	89.8% (84.3–95.2)	9.0%	13.1%	77.9%
72 h	14	51.8% (36.0–67.6)	41.3%	15.3%	43.4%

Note: CIF, cumulative incidence function; KM, Kaplan–Meier.

**Table 5:** Prespecified Cox and competing-risk model for circuit clotting. The cause-specific Cox model and Fine–Gray subdistribution approximation used four clinically prespecified binary predictors and patient-level cluster-robust standard errors.

Model	Predictor	HR/sHR	95% CI	*p* Value	SE Approach
Cause-specific Cox PH (primary)	Femoral catheter vs. non-femoral	1.510	0.604–3.776	0.378	cluster-robust SE
Cause-specific Cox PH (primary)	Platelets < 100 × 10^3^/µL	1.180	0.533–2.613	0.683	cluster-robust SE
Cause-specific Cox PH (primary)	Septic shock	0.835	0.335–2.082	0.698	cluster-robust SE
Cause-specific Cox PH (primary)	Therapeutic anticoagulation indication	1.655	0.689–3.977	0.260	cluster-robust SE
Fine–Gray subdistribution approximation	Femoral catheter vs. non-femoral	1.423	0.576–3.516	0.445	cluster-robust SE
Fine–Gray subdistribution approximation	Platelets < 100 × 10^3^/µL	0.975	0.436–2.179	0.951	cluster-robust SE
Fine–Gray subdistribution approximation	Septic shock	0.736	0.295–1.839	0.512	cluster-robust SE
Fine–Gray subdistribution approximation	Therapeutic anticoagulation indication	2.117	0.866–5.173	0.100	cluster-robust SE

Note: HR, hazard ratio; sHR, subdistribution hazard ratio. All models used 31 circuit clotting events, 223 runs, and 200 patient clusters; estimates are exploratory and should not be interpreted as definitive predictor effects. PH, proportional hazards; SE, standard error; CI, confidence interval; CRRT, continuous renal replacement therapy.

**Table 6:** Sensitivity analyses for circuit clotting. Rows evaluate the effect of the original clotting flag, first-run-only restriction, and exclusion of technical terminations on event rates and 48 h time-to-event estimates.

Sensitivity Analysis	Runs	Events	Event Rate per 100 h (95% CI)	48 h KM Survival (95% CI)	48 h Clotting CIF	Interpretation
Primary cleaned end-reason endpoint, all runs	223	31	0.35 (0.24–0.50)	89.8% (84.3–95.2)	9.0%	Reason = Clotting defines primary circuit clotting.
Original event_filter_clotting flag endpoint, all runs	223	30	0.34 (0.23–0.49)	89.8% (84.3–95.2)	9.0%	Checks impact of one discordant end-reason/event-flag row.
First CRRT run per patient only	200	25	0.32 (0.21–0.47)	90.6% (84.8–96.3)	8.1%	Addresses repeat-run dependence at patient level.
Technical terminations excluded	218	31	0.36 (0.24–0.51)	89.7% (84.2–95.1)	9.1%	Checks whether short technical runs influence estimates.

Note: CIF, cumulative incidence function; CRRT, continuous renal replacement therapy; KM, Kaplan–Meier.

## Data Availability

De-identified data may be made available from the corresponding author on reasonable request, subject to institutional and ethics committee restrictions.
